# Utilization of Public Health Service Increased Risk Donors Yields Equivalent Outcomes in Liver Transplantation

**DOI:** 10.1155/2016/9658904

**Published:** 2016-09-29

**Authors:** V. A. Fleetwood, J. Lusciks, J. Poirier, M. Hertl, E. Y. Chan

**Affiliations:** ^1^Department of General Surgery, Rush University Medical Center, Chicago, IL, USA; ^2^Department of Immunology, Rush University Medical Center, Chicago, IL, USA; ^3^Department of General Surgery, Division of Abdominal Transplantation, Rush University Medical Center, Chicago, IL, USA

## Abstract

*Background*. The PHS increased risk donor (IRD) is underutilized in liver transplantation. We aimed to examine the posttransplant outcomes in recipients of increased-risk organs.* Methods*. We analyzed 228,040 transplants in the Organ Procurement and Transplantation Network database from 2004 to 2013. Endpoints were graft failure and death. Results were controlled for demographics and comorbidities. Statistical analysis utilized Fisher's test and logistic regression.* Results*. 58,816 patients were identified (5,534 IRD, 53,282 non-IRD). IRDs were more frequently male (69.2% versus 58.3%, *p* < 0.001), younger (34 versus 39, *p* < 0.001), and less likely to have comorbidities (*p* < 0.001). Waitlist time was longer for IRD graft recipients (254 versus 238 days, *p* < 0.001). All outcomes were better in the IRD group. Graft failure (23.6 versus 27.3%, *p* < 0.001) and mortality (20.4 versus 22.3%, *p* = 0.001) were decreased in IRD graft recipients. However, in multivariate analysis, IRD status was not a significant indicator of outcomes.* Conclusion*. This is the first study to describe IRD demographics in liver transplantation. Outcomes are improved in IRD organ recipients; however, controlling for donor and recipient comorbidities, ischemia time, and MELD score, the differences lose significance. In multivariate analysis, use of IRD organs is noninferior, with similar graft failure and mortality despite the infectious risk.

## 1. Introduction

The use of liver grafts from Public Health Service (PHS) increased risk donors (IRDs), donors at increased risk for transmission of blood-borne diseases, remains inconsistent in liver transplantation. These donors include those who have been recently incarcerated, those who practice intravenous drug abuse or prostitution, and those with a number of other risk factors [1]. Approximately 8–11% [2] of grafts are categorized as being at increased risk by the 2013 guidelines [1]. Many of these grafts are transplanted late, or not at all: the discard rate is high, and the rate of use of these organs in the renal transplantation literature is less than 70% [3].

The underuse of IRD grafts is in part related to concern for disease transmission. Increased risk donors carry with their graft the possibility of transmission of blood-borne diseases, such as human immunodeficiency virus (HIV), hepatitis C virus (HCV), and hepatitis B virus (HBV). They are by definition seronegative but considered to be at a higher risk of being evaluated in their window period. In surveys and focus group studies, patients report that they are wary of the consequences of viral transmission [4]. Due to the focus on infection, multiple studies [5, 6] have addressed the low rates of recipient infection and the appropriate screening.

However, the organ quality from IRDs and the ultimate effects on survival of IRD use have not been addressed. No data have been published on outcomes of IRD use in liver transplantation to detect whether outcomes differ between increased and average-risk donor organs.

Given the dearth of literature on IRD outcomes in liver transplantation, we examined the UNOS/STAR database. We aimed to examine the differences in outcomes between recipients of increased- and average-risk organs.

## 2. Methods

### 2.1. Data Source and Cohort Identification

Data were deidentified and informed consent was waived. We performed a review of the United Network for Organ Sharing (UNOS)/Standard Transplant and Analysis Research (STAR) files, selecting patients by their unique registration identifiers. Our population was drawn from patients transplanted after 06/30/2004, when IRD data was mandated to be recorded, and before 09/31/2013 to adequately reflect the post-MELD era and to allow at least one year of follow-up. Of note, the guidelines changed in 2013, so our data spans old and new data as well as a brief transition point in which both guidelines could be used; there were insufficient data to isolate the effect of the new guidelines. Inclusion criteria were having received a primary, single-organ liver transplant from an adult deceased donor. Exclusion criteria were multiorgan transplants, having received a previous transplant of any time, and lack of designated IRD or non-IRD status in the UNOS database.

### 2.2. Patient Covariates

We collected information from our database on multiple sociodemographic characteristics including donor and recipient age, sex, and comorbidities. Comorbidities included previous myocardial infarction (MI) and any drug-treated conditions such as hypertension (HTN), diabetes mellitus (DM), and COPD (chronic obstructive pulmonary disease). We also examined specific donor characteristics such as cause of death, race, height, graft type (split versus whole), and share type. Graft data on warm and cold ischemic times were collected and analyzed.

### 2.3. Outcome Measures

Our primary endpoints were graft failure and mortality. Data on graft failure and mortality were complete with 0% missingness.

### 2.4. Statistical Analysis

Statistical analysis was performed in R (version 3.1.1). Statistical significance was set at *p* < 0.05.

#### 2.4.1. Descriptive Analysis

Descriptive analysis compared demographics between groups using Fisher's exact test for binary variables, chi-square analysis for categorical variables, and *t* testing for continuous variables. Fisher's exact test and chi-square testing were used in univariate analysis to evaluate the statistical significance of our primary outcomes between risk groups (IRD versus non-IRD).

To evaluate the unadjusted contributions of continuous factors to risk of graft failure or mortality, continuous variables were stratified into deciles and the unadjusted percentage of graft failure or mortality at each decile plotted.

#### 2.4.2. Multivariate Analysis

We used multivariate logistic regression to evaluate the association between IRD status and our primary endpoints after adjusting for donor, recipient, and graft characteristics. Log rank analysis with Kaplan-Meier survival curves was used to assess survival of grafts and patients.

#### 2.4.3. IRB Approval

Institutional review board approval was obtained prior to initiation of the study. Our study was coded as exempt.

## 3. Results

### 3.1. Donor Demographics

A total of 58,816 eligible records were identified. Of these, 5,536, or 9.4%, were listed as PHS increased risk donors. The two groups, IRD and non-IRD, were compared on age, gender, and prevalence of comorbidities (Table 1).

The IRDs were found to be on average significantly younger, ranging from zero to 92 with a mean age of 34.1 ± standard deviation (SD) 13.4, compared to a non-IRD average of 39.1 ± SD 18.9 (*p* < 0.001). They were also more than 10% more likely to be male (69.23% versus 58.33%, *p* < 0.001). When evaluating health status, the IRDs on average had lower rates of comorbidities: rates of prior myocardial infarction, drug-treated diabetes, and drug-treated hypertension were statistically considerably lower (Table 1). Pressor requirements at the donor operation were lower in the IRD group (51.65% versus 56.55%, *p* < 0.001).

On analysis, the increased risk donors were found to have a lower donor risk index (Table 2). We evaluated the donor risk index (DRI) of each group as described by Feng et al. [7] and found that increased risk donors had an average DRI of 1.64 (±0.39), significantly lower than their non-IRD counterparts (1.87 ± 0.50, *p* < 0.001).

Finally, the differences in groups appeared to be macroscopically apparent in the graft: rates of macrosteatosis were slightly lower in the increased risk group (7.4% versus 8.4%, *p* = 0.002).

### 3.2. Recipient Demographics

Transplant recipients included in our study were similarly classified into IRD recipients and non-IRD recipients and demographics were analyzed. Median follow-up after transplant in the overall population was 33 months. The age of recipients ranged from zero to 83 with a mean age of 52.3 ± 13.3 in IRD recipients and 49.5 ± 17.1 in non-IRD recipients (*p* < 0.001). IRD recipients were, like their donors, significantly more likely to be male (69.82% versus 65.46%, *p* < 0.001). Unlike their donors, they were at statistically equivalent risk of comorbidities: rates of drug-treated diabetes, drug-treated hypertension, and chronic obstructive pulmonary disease were similar between groups. MELD scores were found to be on average marginally higher in the IRD group (21.9 versus 21.4, *p* = 0.002). Graft characteristics were different between groups, with a shorter cold ischemic time in the IRD group (6.92 hours in IRD versus 7.03 hours in non-IRD, *p* < 0.001) but similar warm ischemic time (41.2 minutes in each group, *p* = 0.08).

### 3.3. Graft Failure

We examined rates of graft failure to see if outcomes were equivalent between our groups. On univariate analysis, graft failure was determined to be significantly lower in the IRD group (27.33% non-IRD versus 23.64% IRD, *p* < 0.001).

**Figure 1:**
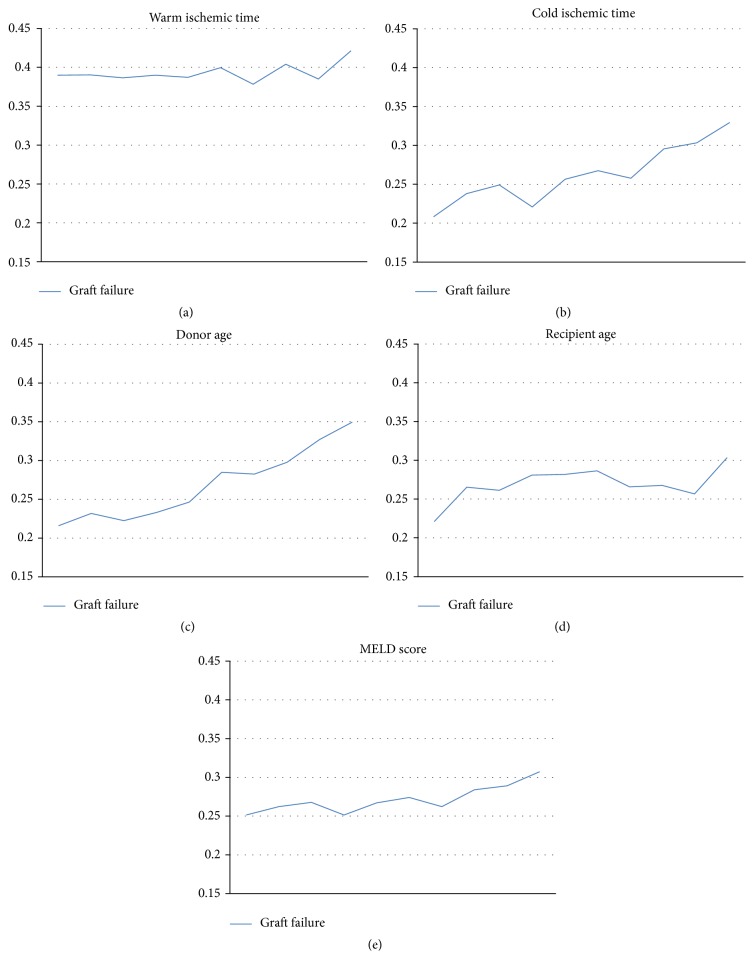
Unadjusted contributions of continuous factors to risk of graft failure. (a) Warm ischemic time (WIT). On multivariate analysis, risk of graft failure increases by 0.2% per minute (*p* = 0.03). (b) Cold ischemic time (CIT) contributes to 2.4% increased graft failure risk per hour. (c) Donor age increases graft failure risk by 0.4% per year of age. (d) Recipient age contributes to 0.5% per year of age. (e) Graft failure rates increase by 0.9% per MELD point.

**Figure 2:**
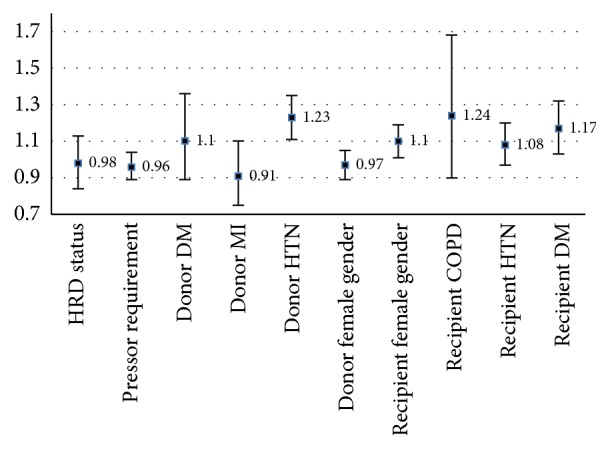
Donor hypertension, recipient female gender, and recipient diabetes mellitus (DM) increase risk of graft failure. When controlling for donor and recipient factors, IRD status (HRD) does not independently impact risk.

When examined on multivariate analysis, increased risk donor status was not protective against graft failure (*p* = 0.74, Figure 2). Not surprisingly, multiple donor and recipient factors and graft ischemic times all contributed to risk of graft failure, as noted in Figures 1 and 2. Most significant among recipients were presence of diabetes (OR 1.6 [CI 1.28–2.01], *p* < 0.001) and recipient female gender (OR 1.09 [CI 1.00–1.09], *p* = 0.03), both of which were independent predictors of risk. Important donor factors were donor age and donor hypertension (OR 1.22 [CI 1.13–1.35], *p* < 0.001), similarly contributing to risk. Increased ischemia time and MELD significantly raised risk as well (Figure 1). Recipient hypertension and COPD as well as donor female gender did not appear to affect rates of graft failure.

### 3.4. Mortality

**Figure 3:**
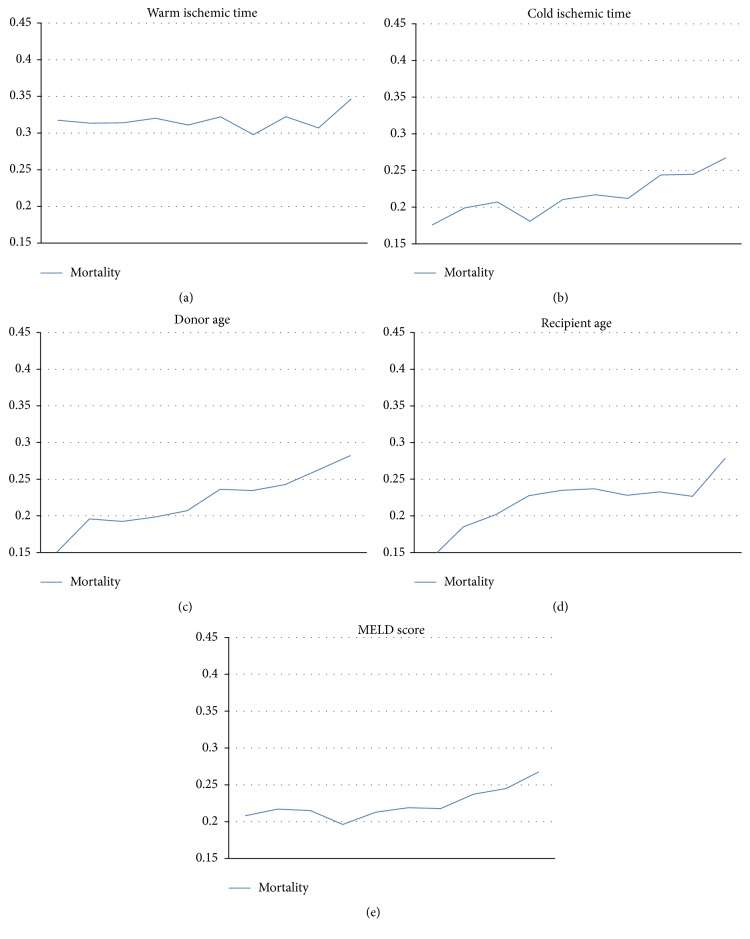
Unadjusted contributions of continuous factors to risk of death. (a) Warm ischemic time (WIT). On multivariate analysis, WIT does not reach significance (*p* > 0.05). (b) Cold ischemic time (CIT) contributes to 2.0% increased graft failure risk per hour. (c) Donor age increases graft failure risk by 1.5% per year of age. (d) Recipient age contributes to 1.7% per year of age. (e) Graft failure rates increase by 1.0% per MELD point.

**Figure 4:**
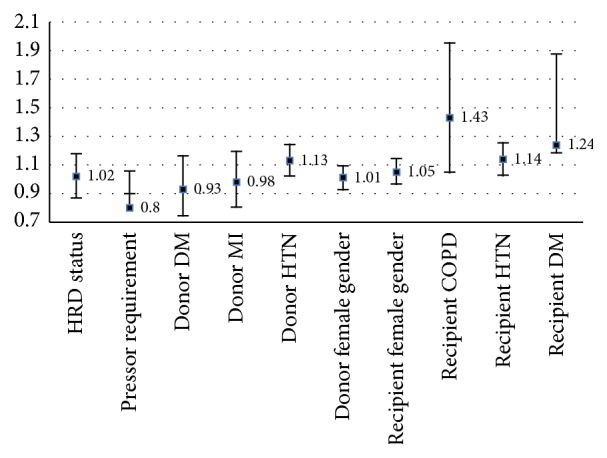
Recipient comorbidities, including DM, COPD, and hypertension (HTN), as well as donor hypertension increase risk of mortality. When controlling for donor and recipient factors, IRD status (HRD) does not independently impact risk.

On unadjusted analysis, mortality was significantly higher in non-IRDs (22.28% versus 20.40%, *p* = 0.001). In adjusted (multivariate) analysis, results were similar to those found with graft failure: IRD status no longer significantly protected against risk when controlling for other variables (*p* = 0.83, Figure 4), and multiple donor, recipient, and graft factors were found to contribute to risk of death (Figures 3 and 4). Most significantly contributing to risk were recipient diabetes (OR 1.49 [CI 1.18–1.88], *p* < 0.001), cold ischemic time (*p* < 0.001, Figure 4), and MELD score (*p* < 0.001, Figure 3). Recipient hypertension and COPD marginally affected mortality. Warm ischemic time reached only borderline significance when analyzing contribution to risk (*p* = 0.05).

## 4. Discussion

Our study demonstrates that, regardless of the risk of disease transmission, clinical outcomes of liver transplantation in IRD-sourced grafts are statistically similar when controlling for recipient and donor characteristics.

We found that donor hypertension, cold ischemic time, and MELD were independent predictors of risk for both graft failure and mortality on multivariate analysis. The increased risk donors were significantly less likely to have hypertension and averaged lower cold ischemia times and MELD scores, likely contributing to the observed lower rates of graft failure and mortality prior to controlling for donor factors and the equalization of outcomes after.

Similarly, recipient comorbidities including hypertension, diabetes, and COPD were mortality risk predictors on our multivariate analysis; diabetes and female gender predicted risk of graft failure. Correspondingly, although recipient comorbidities were similar between groups, IRD recipients were more likely to be male, likely mitigating the effects of female gender on graft failure risk.

Our findings suggest that use of IRD organs leads to noninferior outcomes after transplantation. These similar outcomes allow IRD organs to be a viable source of liver transplants in a persistent nationwide shortage.

No prior studies have addressed the outcomes after liver transplantation using similarly increased risk donors. Although data have addressed the usage patterns of these organs by surgeons [8], none have elucidated the relative risks of graft failure or survival in the population receiving these organs. However, studies in the renal transplantation literature have examined demographic patterns of patients receiving IRD kidneys and have suggested that both donor and recipient characteristics contribute significantly to the outcome of transplantation [9]. Chow et al. in 2013 addressed the varying phenotypes of patients receiving renal transplants and their risk of waitlist mortality, comparing this to the risk of death after seroconversion [9]. The results established the contribution of multiple demographic variables to each phenotype and showed that certain patients benefit from receiving IRD organs, while others have an unfavorable risk/benefit ratio based on age, PRA, and other variables. We believe our data provides the background necessary to address which variables affect mortality and allows a springboard for the development of graft failure and survival nomograms.

Although we discuss graft failure and survival data, our study intentionally does not calculate the rates of seroconversion in our population. Our purpose is to provide information on outcomes and risk factors for these outcomes; we hope to allow both transplant providers and patients to feel more at ease with the informed consent for an increased risk donor organ. However, an equivalent risk of graft or patient complications does not mitigate the concern that we may inadvertently transmit infection with the use of increased risk grafts. All providers must pursue a frank discussion with the patient about the risks and benefits of IRD organ use. A special informed consent should be used, as it both informs the patient more fully and is associated with increased utilization of IRD organs [10]. A thorough discussion should emphasize the exceedingly low risk of viral transmission [3, 4] but also require the patient to express understanding of the need for close surveillance after transplantation, as early recognition of infectious complications can allow early intervention in other recipients [11]. Furthermore, the patient should be aware that a substantial amount of waitlist mortality results from declined livers, rather than lack of opportunity for transplantation [12], and that in carefully selected patients an IRD graft may carry a lower chance of death than declining an organ [13]. An educated patient-provider discussion should be individualized to the patient, their time on the waitlist, and their likelihood of receiving another offer, as all of these have been found to be helpful in identifying the ideal recipient of an IRD graft [9].

Interestingly, outcomes are similar despite the risk of infectious transmission. Therefore, the risk of infection has not caused higher rates of graft failure and mortality. The observed outcomes can contribute to an informed patient discussion of risk.

Our study has certain limitations that must be addressed. Chief among these is the retrospective nature of the study, limiting true assessment of causation. Furthermore, the UNOS database is based on organization self-reporting and thereby highly susceptible to user error; some of the provided data may be incomplete or frankly inaccurate.

Another limitation is the choice of data in our multivariate model. We selected the demographic and health data used as variables in our analysis based on a panel discussion which found this data to be the most likely to contribute to outcomes. However, in isolating certain variables from the database, we may have missed others that contribute significantly to risk. Also, we believe the inclusion of multiple variables in our analysis without requiring significance on univariate analysis is necessary, to fully control for confounders; but this may cause related factors to lose significance when too closely correlated in the logistic regression.

Finally, given the risky behaviors exhibited by the PHS increased risk donors, medical follow-up may have been limited, and the reported rates of comorbidities may have been artificially low, leading to the IRD population appearing healthier than it is. However, with such a high-powered study, we feel that we have been able to capture enough data that any small inconsistencies would not significantly affect the measured outcomes.

## 5. Conclusion

Transplantation of liver grafts from donors classified as PHS increased risk is associated with similar posttransplant outcomes, including similar rates of graft failure and mortality. Although the infectious risk, albeit rare, remains, patients should be counseled extensively on the benefits of proceeding with transplantation, the risks of remaining on the waitlist, and the procedure to be followed should they seroconvert. With the constant waitlist mortality, the benefits of utilizing increased risk donor organs cannot be ignored.

## Figures and Tables

**Table 1:** Donor demographics.

Variable	IRD		Non-IRD	
	*n*	%	*n*	%
Age				
<40	3676	66.40%	25626	48.10%
40–49	1052	19.00%	9617	18.05%
50–59	596	10.77%	10017	18.80%
60–69	174	3.14%	5697	10.69%
70+	38	0.69%	2323	4.36%
Cause of death				
Anoxia	1932	34.91%	10460	19.63%
CVA	1218	22.01%	21072	39.55%
Other	2384	43.08%	21750	40.82%
Race				
African American	941	15.73%	9200	17.41%
White	3894	65.08%	34742	65.76%
Hispanic	611	10.21%	7305	13.83%
Asian	53	0.89%	1282	2.43%
Am. Indian/Alaskan	14	0.23%	198	0.37%
Pacific Islander	5	0.08%	90	0.17%
Multiracial	16	0.27%	465	0.88%
Donor type				
DCD	269	4.86%	2393	4.49%
DBD	5265	95.14%	50888	95.51%
Graft type				
Split	133	2.40%	1931	3.63%
Whole	5401	97.60%	51306	96.37%
Donor height	172.4 (±14.6)		167.1 (±21.3)	
Share type				
Local	3858	69.71%	37193	69.81%
Regional	1285	23.22%	12536	23.53%
National	391	7.07%	3545	6.65%
Donor risk index	1.64 (±0.39)		1.87 (±0.50)	

**Table 2:** Donor and recipient comorbidities.

Comorbidity	IRD		Non-IRD		*p*
	*n*	%	*n*	%	
Hypertension					
Donor	1217	82.45%	17183	86.24%	<0.001
Recipient	259	17.55%	2742	13.76%	0.14
Diabetes					
Donor	330	20.45%	5358	31.15%	<0.001
Recipient	1284	79.55%	11845	68.85%	0.1
Donor MI	114		1951		<0.001
Recipient COPD	18		215		0.98

## Abbreviations

CI: 95% confidence interval
COPD: Chronic obstructive pulmonary disease
DM: Diabetes mellitus
HBV: Hepatitis B virus
HCV: Hepatitis C virus
HIV: Human immunodeficiency virus
HTN: Hypertension
IRB: Institutional review board
IRD: Increased risk donor
MI: Myocardial infarction
MELD: Model for end stage liver disease
OLT: Orthotopic liver transplantation
OR: Odds ratio
STAR: Standard transplant and analysis research
UNOS: United Network for Organ Sharing.
